# Diffuse Alveolar Hemorrhage Secondary to Acute Mitral Regurgitation

**DOI:** 10.14797/mdcvj.1152

**Published:** 2022-12-09

**Authors:** Fouad Khalil, Jaswanth R. Jasti, Mohammad Ali, Huthayfa Ateeli

**Affiliations:** 1 University of South Dakota, Sioux Falls, South Dakota, US; 2 Avera Hospital, Sioux Falls, South Dakota, US

**Keywords:** diffuse alveolar hemorrhage, acute mitral regurgitation, echocardiography, mitral valve surgery

## Abstract

We describe acute mitral valve regurgitation in a young, previously healthy male patient presenting with diffuse alveolar hemorrhage. The patient initially presented with acute respiratory failure with refractory arterial hypoxemia despite mechanical ventilation. Bronchoscopy showed diffuse alveolar hemorrhage. The patient quickly developed cardiogenic shock, which required vasopressor infusion. Echocardiography showed severe mitral regurgitation and myxomatous mitral valve with anterior leaflet prolapse along with chordal rupture involving the anterior leaflet, which was flail. An Impella device was emergently placed, and the patient underwent mitral valve replacement with subsequent quick resolution of all symptoms.

## Background

Acute mitral regurgitation (MR) usually presents with hemodynamic instability that requires urgent management. Early diagnosis is critical, as presentation might mimic acute pulmonary processes.^1^ Acute MR commonly presents with sudden-onset pulmonary edema and cardiogenic shock. Rare presentations such as hemoptysis and diffuse alveolar hemorrhage are possible. The increased atrial pressure and the decreased systemic pressure might diminish MR murmur. Therefore, the absence of systolic murmur does not exclude acute MR. Echocardiography is the first choice of imaging modalities.^2^ Temporary stabilization with a short-term mechanical assist device is usually required before surgery. According to the 2020 American College of Cardiology guidelines, urgent surgery is indicated in patients with acute severe MR, and valve replacement is generally required in the case of papillary muscle rupture.^3^

## Case report

### Patient Presentation

**Figure 1:**
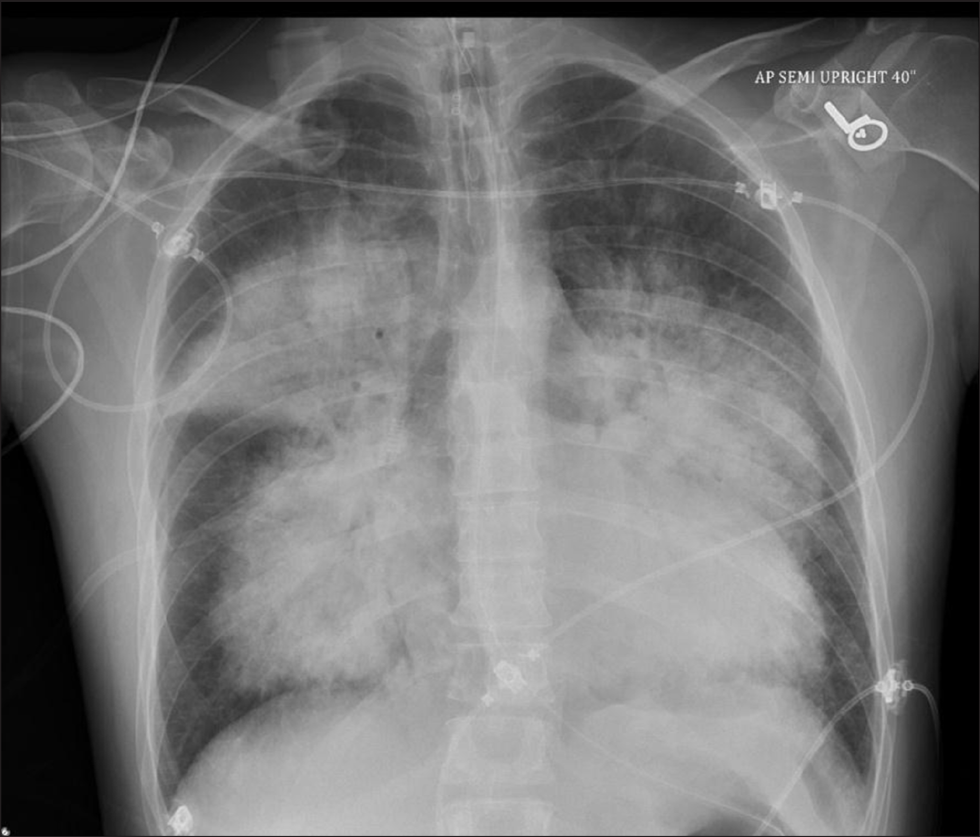
Chest x-ray upon presentation showing extensive dense perihilar mass-like consolidative opacities.

A 36-year-old male presented to the emergency department (ED) with a 2-day history of cough and shortness of breath. The patient did not have any past medical or surgical history. His shortness of breath started suddenly and had been progressively worsening over the past 2 days. His cough was initially dry but became associated with blood streaks on the day of presentation. In the ED, vital signs showed body temperature of 37.9°C, pulse rate of 150 beats/minute, and respiratory rate of 41 breaths/minute. Oxygen saturation (SpO2) was 68% on room air. The physical exam was remarkable for diffuse crackles over lung fields bilaterally. No appreciable cardiac murmurs were detected. The chest x-ray scan revealed extensive dense perihilar mass-like consolidative opacities (Figure 1).

### Initial Work-Up

**Figure 2:**
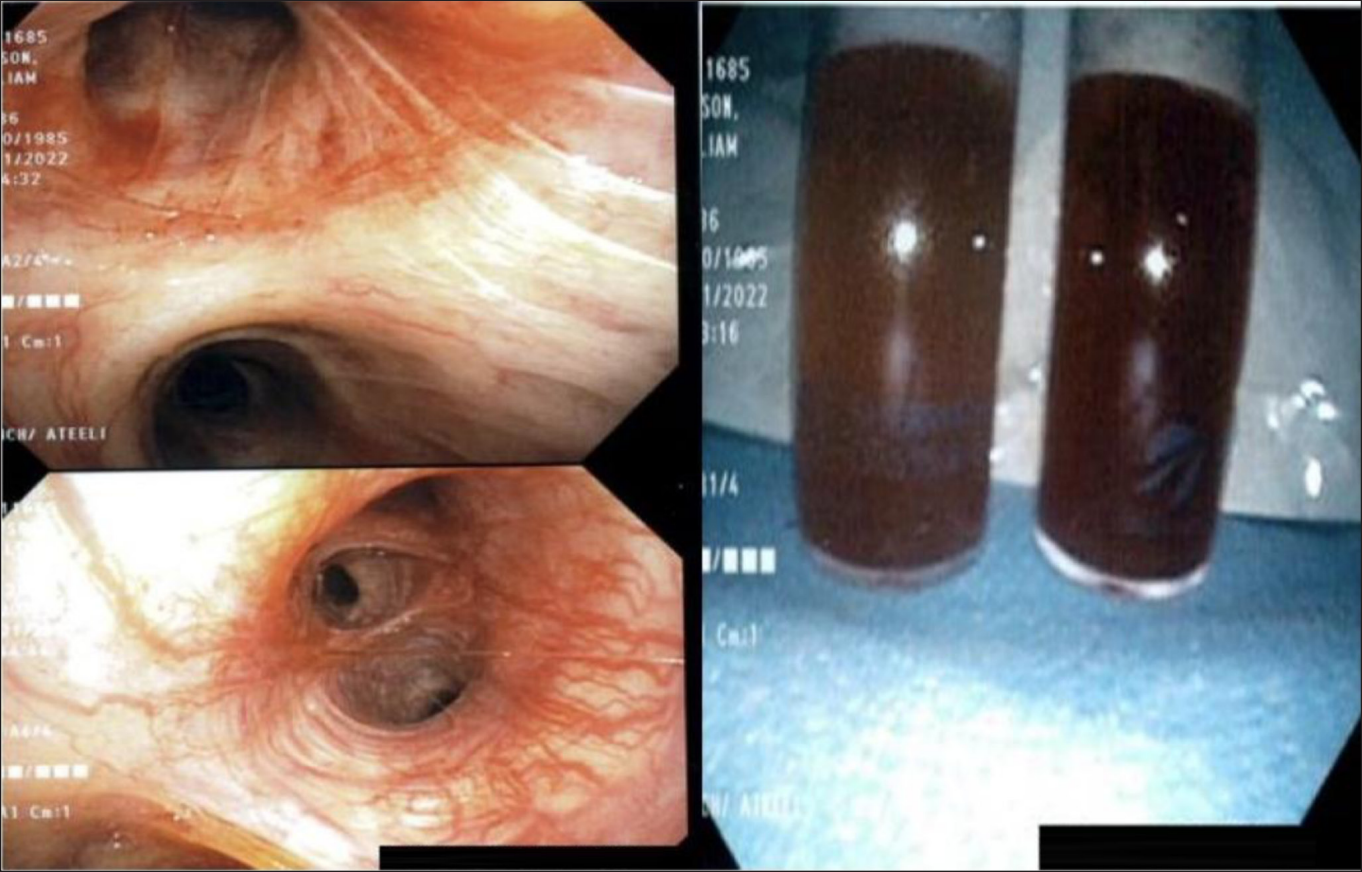
Bronchoscopy showing alveolar hemorrhage.

An electrocardiogram (ECG) showed sinus tachycardia. Labs were notable for white blood counts of 32,000 K/uL (reference 4000–11000 K/uL), lactic acid 3.3 mmol/L (reference 0.5–2 mmol/L), high-sensitivity troponin of 128 pg/mL (reference < 20 pg/mL), and B-natriuretic peptide of 494 pg/mL (reference < 100). Arterial blood gas showed a pH of 7.19. PCO_2_ 60 and PO_2_ 42 mm Hg. The patient was started on supplemental oxygen in the ED and was titrated up to bilevel positive airway pressure without significant improvement of SpO2. The patient was transferred to the intensive care unit, intubated, and started on mechanical ventilation for worsening respiratory failure and altered mental status. SpO2 was in the 80s despite mechanical ventilation with high positive end-expiratory pressure. Bronchoscopy showed diffuse alveolar hemorrhage (Figure 2).

**Figure 3:**
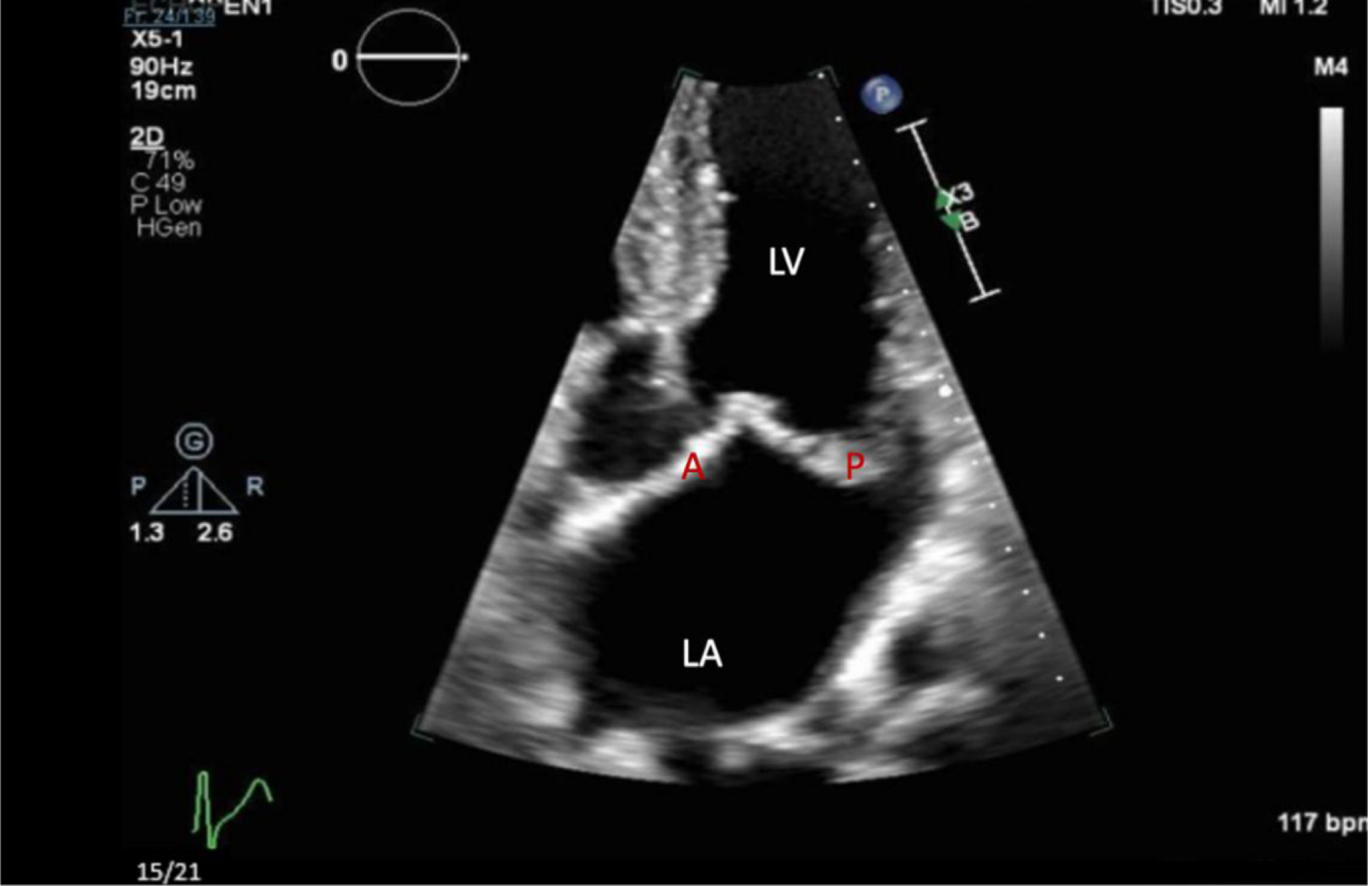
Zoomed in transthoracic echocardiogram with apical four-chamber view showing the left ventricle (LV), left atrium (LA), posterior (P), and anterior (A) mitral valve leaflets.

**Figure 4:**
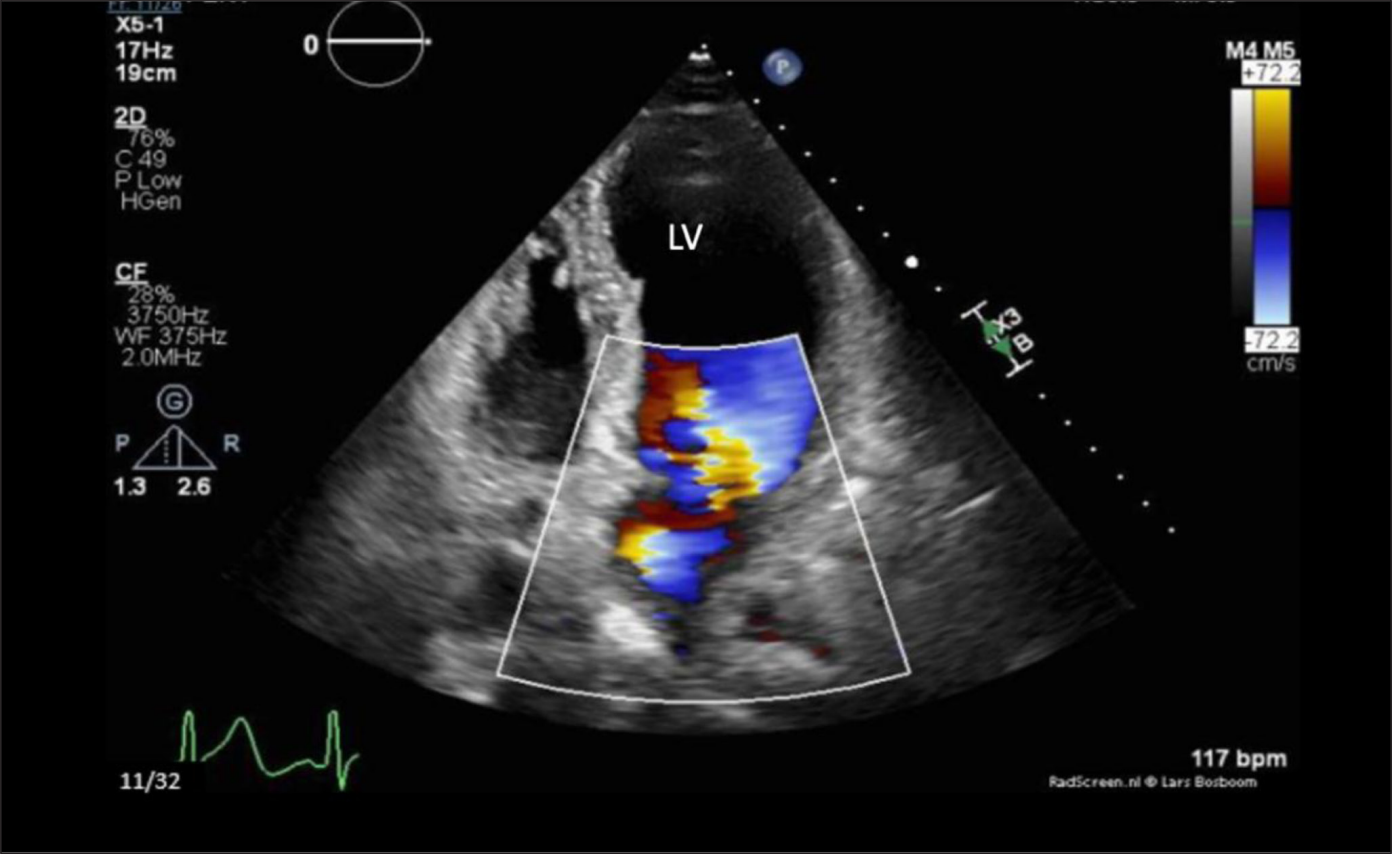
Transthoracic echocardiogram with apical four-chamber view in systole, showing severe jet of mitral valve regurgitations.

Intravenous norepinephrine infusion was initiated for blood pressure support. Transthoracic ECG showed severe mitral regurgitation (Figures 3, 4).

**Figure 5:**
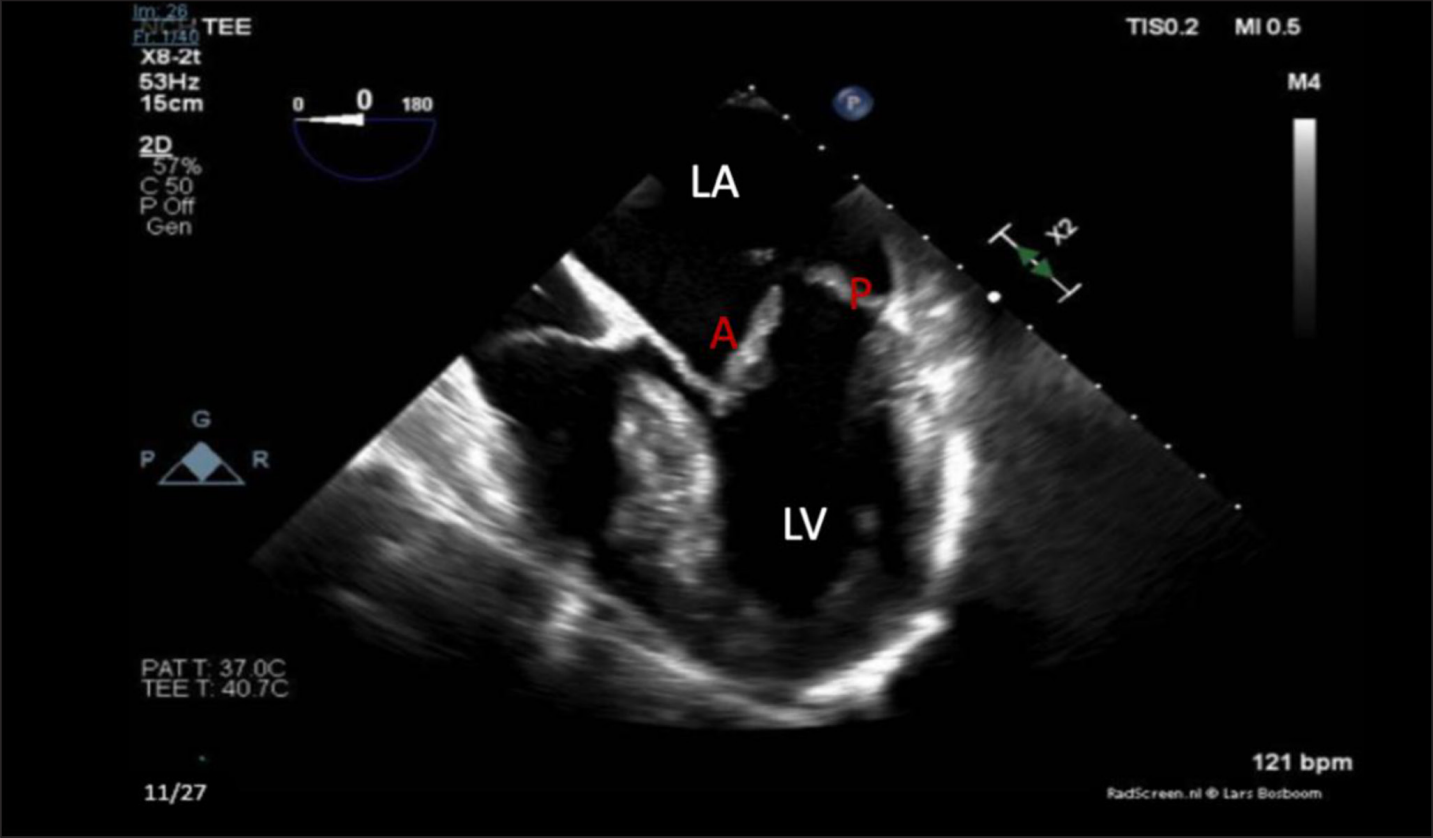
Mid-esophageal transesophageal echocardiogram with 4-chamber apical view view showing left ventricle (LV) and left atrium (LA). Myxomatous degeneration and severe prolapse of the anterior and posterior leaflets of the mitral valve are seen on this image.

**Figure 6:**
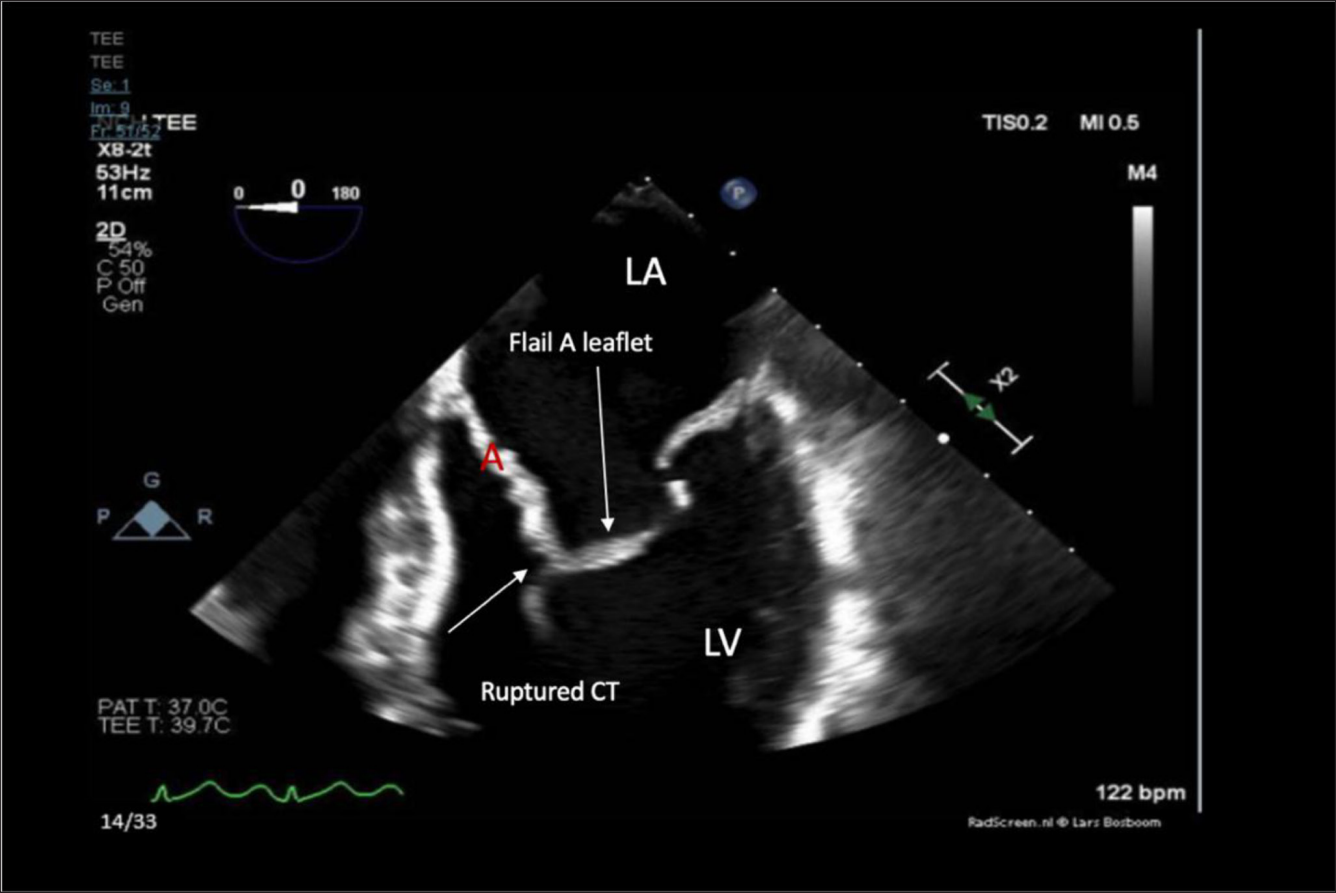
Zoomed in mid esophageal transesophageal echocardiogram with 4-apical view showing left ventricle (LV), left atrium (LA), chordae tendineae (CT), and flail (A) mitral valve leaflet.

Transesophageal ECG showed a myxomatous mitral valve with anterior leaflet prolapse along with chordal rupture involving the anterior leaflet, which was flail (Figures 5, 6; Videos 1, 2, 3). A posteriorly directed jet of severe mitral regurgitation was noted as well.

**Video 1:** Transesophageal echocardiograms showing a myxomatous mitral valve with anterior leaflet prolapse along with chordal rupture involving the flail anterior leaflet, see also at https://youtu.be/J6KtVU3eSNk.

**Video 2:** Color Doppler transesophageal echocardiogram showing the mitral valve with flail anterior leaflet and severe mitral regurgitation, see also at https://youtu.be/pSyQjq4Xm4A.

**Video 3:** Zoomed 3-dimensional transesophageal echocardiogram view of the myxomatous mitral valve showing the flail anterior leaflet, see also at https://youtu.be/d1v8SeTqmmE.

### Diagnosis and Management

Cardiothoracic surgery was consulted for acute severe mitral regurgitation with chordal rupture. The patient was taken emergently to the operating room, where an Impella device (Abiomed) was placed with subsequent improvement of oxygenation and blood pressure. The coronary angiography showed normal coronary arteries. During surgery, the surgeon discovered very large A2 and P2 scallops that were completely unsupported as well as the valve itself being rotated counterclockwise from its normal position. Due to the patient’s complex degenerative disease of the mitral valve, the On-X mechanical mitral valve (CryoLife, Inc.) was placed according to the preserved A3 scallop and posterior leaflet after removing the A2 scallop and most of the ruptured chordae. There were no periprocedural complications.

### Follow-up

On postoperative day 2, the patient was extubated and weaned off vasopressors. After a few days of diuresis with intravenous frusemide, the patient was weaned off oxygen completely. He was discharged home on warfarin 7 days after the surgery. On a 4-week follow-up visit, the patient was asymptomatic, doing well, and within the targeted therapeutic international normalized ratio range. The pathology of the excised mitral valve tissue revealed valvular connective tissue with fibrosis and degenerative/myxoid changes, but no significant acute inflammation or vegetations.

## Conclusion

Acute mitral regurgitation is a medical and surgical emergency. Diagnosis could be challenging as patients frequently do not have any cardiac history and oftentimes present with symptoms resembling primary lung processes. Providers should be aware of rare presentations such as hemoptysis and diffuse alveolar hemorrhage. Echocardiography is the first-choice imaging modality for diagnosis. Urgent mitral valve surgery is usually the definitive treatment.
